# A multidisciplinary experimental and methodological investigation of electron and proton minibeams in the framework of the INFN MIRO project

**DOI:** 10.1002/mp.70537

**Published:** 2026-08-03

**Authors:** Francesco Romano, Francesca Cella Zanacchi, Esther Ciarrocchi, Gaia Franciosini, Giuliana Milluzzo, Emanuele Scifoni, Anna Vignati, Said Ahmad, Silvia Arezzini, Andrea Attili, Marco Battestini, Jacopo Bernardini, Matteo Bettanin, Alessandra Bisio, Maria Giuseppina Bisogni, Igor Bodrenko, Giulio Bordieri, Valentina Bravatà, Alberto Burattini, Massimo Camarda, Francesco Paolo Cammarata, Simone Capaccioli, Lorenzo Castelli, Andrea Cavalieri, Mariagrazia Celentano, Enrica Chiadroni, Francesco Giuseppe Cordoni, Elena Corvaia, Mario Costa, Alessandro Curcio, Eleonora Da Pozzo, Martina De Felice, Francesca Del Debbio, Francesco D'Errico, Maria Cristina D'Oca, Beatrice D'Orsi, Umberto Deut, Elisabetta Durisi, Maria Evelina Fantacci, Stefano Farina, Giuseppe Felici, Arianna Ferro, Luca Ficcadenti, Antonino Formuso, Giusi Irma Forte, Simona Giordanengo, Alessia Giuliano, Lucia Giuliano, Luca Lanzanò, Stefania Linsalata, Simone Lossano, Margherita Maffei, Michela Marafini, Maurizio Marrale, Maurizio Massa, Luigi Masturzo, Enrico Mazzoni, Mauro Migliorati, Felix Mas Milian, Luigi Minafra, Andrea Moggi, Diango Montalvan Olivares, Marco Montefiori, Matteo Morrocchi, Andrea Mostacci, Franco Mostardi, Chinonso Okpuwe, Fabiola Paiar, Luigi Palumbo, Vincenzo Patera, Jake Harold Pensavalle, Gaia Pucci, Flaminia Quattrini, Alessandra Retico, Valeria Rosso, Giorgio Russo, Roberto Sacchi, Alessio Sarti, Silvia Scalisi, Camilla Scapicchio, Angelo Schiavi, Paolo Stochino, Enrica Strettoi, Francesco Tommasino, Valentina Tozzini, Giacomo Traini, Gabriele Trovato, Alice Usai, Francesco Urso, Fabio Di Martino

**Affiliations:** ^1^ Catania Division National Institute of Nuclear Physics (INFN) Catania Italy; ^2^ Department of Physics “E. Fermi” University of Pisa Pisa Italy; ^3^ Center for Instrument Sharing University of Pisa (CISUP) Pisa Italy; ^4^ Nanoscopy and NIC@IIT Istituto Italiano di Tecnologia Genova Italy; ^5^ Pisa Division National Institute of Nuclear Physics (INFN) Pisa Italy; ^6^ Dipartimento di Scienze di Base applicate per l'ingegneria Sapienza Università di Roma Rome Italy; ^7^ Roma I Division National Institute of Nuclear Physics (INFN) Rome Italy; ^8^ Trento Institute for Fundamental Physics and Applications (TIFPA) National Institute for Nuclear Physics, (INFN) Povo Italy; ^9^ Department of Physics University of Trento Povo Italy; ^10^ Physics Department Università degli Studi di Torino Torino Italy; ^11^ Torino division National Institute for Nuclear Physics (INFN) Torino Italy; ^12^ Department of Physics University of Messina Messina Italy; ^13^ Rome 3 division National Institute for Nuclear Physics (INFN) Rome Italy; ^14^ Department of Cellular, Computational and Integrative Biology University of Trento Torino Italy; ^15^ Centro Pisano multidisciplinare sulla ricerca e implementazione clinica della Flash Radiotherapy (CPFR) CPFR@CISUP facilities Pisa Italy; ^16^ Department of Physics University of Cagliari, Cittadella Universitaria di Monserrato Monserrato Cagliari Italy; ^17^ Istituto Nanoscienze‐CNR NEST‐SNS Piazza San Silvestro Pisa Italy; ^18^ Institute of Bioimaging and Complex Biological Systems‐National Research Council (IBSBC‐CNR) Cefalù Italy; ^19^ Laboratori Nazionali del Sud, INFN‐LNS National Institute for Nuclear Physics Catania Italy; ^20^ Sezione di Catania Istituto per la Microelettronica e Microsistemi CNR‐IMM Catania Italy; ^21^ STLab srl Catania Italy; ^22^ Laboratori Nazionali di Frascati INFN Trento Italy; ^23^ Department of Civil Environmental and Mechanical Engineering University of Trento Trento Italy; ^24^ Dipartimento di Fisica e Chimica Emilio Segré Università degli Studi di Palermo Palermo Italia; ^25^ Institute of Neuroscience Italian National Research Council Pisa Italy; ^26^ Department of Pharmacy University of Pisa Pisa Italy; ^27^ Department of Civil and Industrial Engineering University of Pisa Pisa Italy; ^28^ SIT S.p.A. Aprilia, Latina Italy; ^29^ U.O. Fisica Sanitaria Azienda Ospedaliero‐universitaria pisana Pisa Italy; ^30^ Department of Physics and Astronomy “Ettore Majorana” University of Catania Catania Italy; ^31^ Institute of Clinical Physiology Italian National Research Council Pisa Italy; ^32^ Museo Storico della Fisica e Centro Studi e Ricerche ”E. Fermi” Roma Italy; ^33^ Department of Exact and Technological Sciences Universidade Estadual de Santa Cruz Ilhéus Brazil; ^34^ Radiation Oncology Unit, Department of Translational Research and New Technologies in Medicine and Surgery Pisa University Hospital “Azienda Ospedaliero‐Universitaria Pisana” Pisa Italy; ^35^ Cagliari Division National Institute of Nuclear Physics (INFN) Cagliari Italy

## Abstract

**Background:**

The clinical translation of Minibeam RT (MBRT) has recently started thanks to the first human treatments recently performed. However, despite experimental evidence, the impact of the dose distribution parameters involved on the magnitude of the effect itself and the underlying radiobiological mechanisms are still only partially understood. To address this issue, systematic investigations are needed through the implementation of advanced quantitative experiments with a multidisciplinary approach, which is the one proposed in the framework of the INFN funded MIRO (MInibeam RadiOtherapy) project.

**Purpose:**

The aim of this work is to report on the on‐going main activities recently carried out in the framework of the project, showing some of the main results achieved during the first 2 years of the project, in terms of: (i) facilities development and characterization; (ii) new dosimetric approaches; (iii) biological investigation of the effect; and (iv) development of the first tools for dose planning. The multidisciplinary approach adopted by this national Collaboration to tackle the main challenges of minibeam radiotherapy for a reliable and solid clinical translation is discussed.

**Methods:**

The facilities involved in the project are: (i) two facilities dedicated to low (up to 9 MeV) and medium (up to 18 MeV) energy electron minibeam studies, one of them also equipped with an electron FLASH LINAC to study possible synergistic effects with UHDR beams; (ii) one facility dedicated to proton minibeam studies, with energies from 70 to 140 MeV. Novel dosimetric approaches, are presented, mainly based on scintillators, silicon and silicon carbide detectors. Novel techniques for the analysis of biological samples are described, leveraging both integrated averaged data and spatially resolved analysis. Furthermore, a framework for biological modeling using a multiscale approach is presented. A dedicated dose‐planning tool is currently under development to compare “virtual” minibeam plans with conventional ones and, consequently, to quantitatively investigate the potential for clinical translation.

**Results:**

All the facilities were dosimetrically characterized, demonstrating the capability of producing controlled and reproducible electron and proton minibeams. These beams exhibited diverse physical parameters, including peak‐to‐valley ratios between 3 and 30 at the entrance and center‐to‐center distances between 2 and 3 mm. The developed detectors, specifically scintillators and silicon detectors, successfully measured the minibeam patterns with sub‐millimeter resolution, while large‐area silicon carbide detectors were used for average dose measurements. First in‐vitro biological investigations performed with low energy minibeams clearly showed an enhanced survival fraction in the 16HBE healthy lung cells, while maintaining iso‐effective cell killing in A549 cancer cells. Furthermore, a synergy was observed when combining UHDR electron beams with minibeams.

**Conclusions:**

The multidisciplinary approach was consolidated during these first 2 years of the MIRO project, as demonstrated by the results obtained both in terms of dosimetric characterization and biological investigations. The dedicated framework for modelling is being optimized to support the biological findings, allowing for a better interpretation of the results. The tool for dose planning, still under development, will allow the investigation of peculiar configurations to explore new frontiers in the perspective of future human trials.

## 1 INTRODUCTION

Delivering radiation dose to tumors for radiation treatments is limited by potential harm to surrounding organs.^1^ In particular, the treatment of some radioresistant tumors close to sensitive organs (such as the spinal cord), local advanced tumors in critical organs (brain, lungs), and pediatric cancers are still compromised due to the severe side effects to healthy tissues. Spatially Fractionated Radio Therapy (SFRT) consisting on delivery a non‐uniform spatial dose distribution, is certainly a distinctive radiation approach with respect to the conventional uniform radiotherapy leading to a significative impact in increasing the therapeutic index in RT as observed in.^2^ The Minibeam RT (MBRT) which makes use of sub‐millimetric parallel beams (0.5–1 mm) spaced in 1–3 mm represents an achievable good compromise between feasibility of the GRID (cm) and efficacy of the microbeam radiotherapy (MRT) (<100 um) approach with a realistic clinical perspective.^3^, ^4^ Indeed, minibeams of about (or less) 1 mm size still induce a remarkable reduced toxicity in normal tissues which exhibits relevant prompt vascular repair in normal tissues and a superior tumor control with improved long‐term survivals in gliosarcoma bearing rats.

The first treatments with minibeams, although still palliative, are on‐going as reported in^5^ and future trials are planned, further emphasizing how crucial is now a systematic characterization of the minibeam effect for a full understanding of the mechanism behind and a reliable control of the treatments from the dosimetric point of view.^6^

To date, the main hypotheses regarding the radiobiological effects of minibeams take into account multiple multiscale mechanisms, from the cellular to the tissue level.^7^ In particular, these mechanisms appear to be involved: the production of ROS and their recombination,^8^ by‐stander and abscopal effects^9^ (Asur, R., et al., High dose bystander effects in spatially fractionated radiation therapy. Cancer Lett, 2015. 356(1): p. 52–7), immune activation^10^ and vascular response.^11^ Regarding the role of minibeam pattern parameters, there is experimental evidence of the relevant role of both peak dose^12^, ^13^ and valley dose^14^, ^15^, ^16^: in particular, this last parameter appears to play a fundamental role in both tumor control and normal‐tissue toxicity, as shown in literature.^12^, ^13^

Despite a big effort was put so far on studying the correlation between the mentioned physical parameters and the biological response, according to the evidences shown in the cited papers, the impact of the dose distribution parameters involved on the magnitude of the effect itself and the underlying radiobiological mechanisms are still only partially understood. Further, the quantification itself of the sparing effect, is also getting a poor consensus to date. In this context a recent proposition was to introduce a Biologically effective particle number ratio.^17^ To address these open issues, further systematic investigations are needed through the implementation of advanced quantitative experiments with a multidisciplinary approach, involving: (i) experimental setups capable of independently varying parameters over a wide range; (ii) accurate dosimetric systems capable of measuring them in a reliable and reproducible way; (iii) advanced biological and biophysical techniques capable of sensitively appreciating variations in the radiobiological effect both integrated across the entire sample and along the spatial pattern of dose distribution; and (iv) theoretical‐computational modeling that, with continuous feedback from experimental data, formulates and tests new hypotheses and provides guidance for new experiments.

The MIRO (MInibeam RadiOtherapy) project, funded by the INFN Fifth Committee for Technological and Interdisciplinary Physics (CSN5) and started in 2024, is conceptually structured in four work‐packages (WP) characterized as follows:

‐ WP1: Development of experimental platforms capable of producing minibeams with different particle types (protons and electrons) at different energies (9 and 18 MeV electrons, >70 MeV for protons) to systematically investigate the dependencies. One of these platforms, that is, the 9 MeV electron platform at the Centro Pisano for Flash Radiotherapy (CPFR), also allows for the generation of both minibeams in conventional low dose rate and UHDR minibeams, thus also allowing for the investigation of possible synergy between the FLASH and the mini beam radiobiological effect.
‐ WP2: Development and characterization of dosimetric systems and detectors allowing the determination and measurement of all the dosimetric parameters of interest in the minibeam context, including: (i) reconstruction of the relative dose pattern distribution to calculate the radiobiological parameters of interest (c‐t‐c and peak size, PVDR, dose at the peak and dose at the valley); (ii) calculation and measurement of the average integral dose, to be used as a normalization value for correctly comparing the results of minibeam irradiations with the ones obtained with the conventional uniform dose distribution.
‐ WP3: Development of: (i) new methodological approaches in radiobiological in‐vitro and in‐vivo experiments, capable of sensitively studying (resolving small variations) the radiobiological response not only globally but also along the minibeam spatial pattern; (ii) development of multi‐scale theoretical‐computational modelling systems, capable of formulating and testing hypotheses on the mechanisms underlying the effect, continuously feedbacked with experimental results.
‐ WP4: Development of virtual planning systems, investigating the clinical perspectives of the mini‐beam effect and its possible synergistic action with the FLASH effect, through quantitative comparisons, in terms of Relative Biological Effectiveness (RBE), with plans performed using conventional techniques.

In this paper, we will present the conceptual design, the status and the first results of the MIRO project, regarding the dosimetric characterization at the different facilities with both electron and proton minibeam, and the first radiobiological in‐vitro investigations. As for the latter, we report new experimental data on lung cell lines. Given the limited efficacy of conventional radiotherapy for locally advanced thoracic tumors, there is a significant need to explore alternative approaches. Specifically, we discuss the potential of spatially fractionated radiotherapy, including its combination with FLASH radiotherapy, in the final part of this work.

## 2 MATERIALS AND METHODS

### 2.1 Minibeam production

The main aim of the MIRO project is to produce reliable multidisciplinary and variable platforms delivering minibeams for radiobiological, in vitro and in vivo irradiations to systematically investigate the minibeam effect.

The following facilities are available and have been modified to produce minibeams: (i) conventional and UHDR pulsed e‐beams (7–9 MeV) at the CPFR facility in Pisa;^18^ (ii) Conventional and UHDR pulsed e‐beams (10–18 MeV) at the LINAC at the Physics Department in Turin;^19^ (iii) Conventional (low dose rate) proton beams accelerated by an isochronous 9cyclotron (70 or 148 MeV) at the Proton Therapy Centre in Trento.^20^

The chosen approach for minibeam production relies on the use of passive tungsten minibeam collimators with different configurations in terms of hole size and c‐t‐c distance and geometry, optimized for each of the facilities available in the project. Tungsten was chosen as material for all the collimators following the previous study reported in^21^ as it allows to maximize the PVDR minimizing the septal penetration as respect to plastic materials.

Monte Carlo codes, using Geant4^22^ (CPFR), EGS^23^ (CPFR) and TOPAS^24^ (Turin and Trento), reproducing the three facilities and the minibeam generation have been accurately implemented to provide tools providing the prediction of the experimental minibeam outcome^19^, ^21^

Pensavalle et al.^21^ already reported the full dosimetric characterization of the realized minibeam collimators performed with the 9 MeV electron beams available at the CPFR, showing the capability of the facility to obtain high PVDR (15–30) at the surface (0 mm). Moreover, the flexibility of the Linac at the CPFR, which allows for beam current variation within a single pulse, enables a wide range of irradiation conditions. This includes diverse doses per pulse, as well as instantaneous and average dose rates, fulfilling a key requirement for a systematic investigation of the potential synergy between FLASH and minibeam effects.

Table 1 reports the geometrical configurations of the minibeam collimators normalized at the CPFR and also used for preliminary dosimetric studies at the Linac in Turin and to produce proton minibeams at the Trento Protontherapy Beam Line (TPBL).

**TABLE 1:** Minibeam collimators details. The used labels stand for Planar/GRID [Hole width]_[c‐t‐c]_[Field side].

Configuration	Hole width	c‐t‐c	Field	Label
Planar	1 mm	3 mm	20 × 20 mm^2^	*Planar1_3_2*
GRID	1 mm	3 mm	20 × 20 mm^2^	*GRID1_3_2*
Planar	1 mm	3 mm	50 × 50 mm^2^	*Planar1_3_5*
Planar	1 mm	2 mm	50 × 50 mm^2^	*Planar1_2_5*
Planar	0.5 mm	2 mm	10 × 10 mm^2^	*Planar0.5_2_1*
GRID	0.5 mm	2 mm	10 × 10 mm^2^	*GRID0.5_2_1*

**FIGURE 1:**
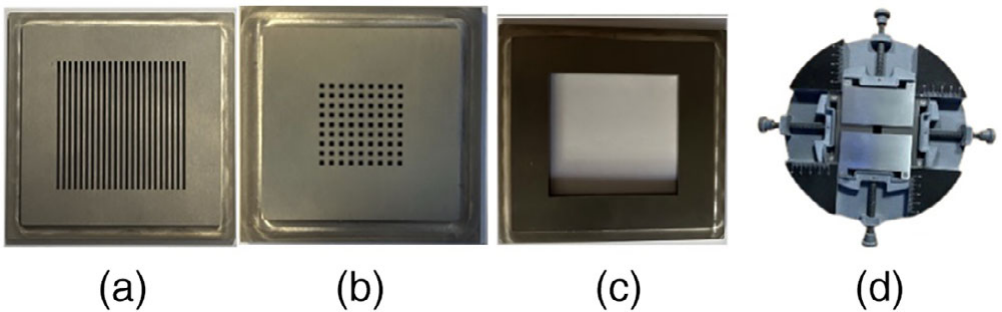
Planar (a), GRID (b) and open field (c) templates with different c‐t‐c distances and (d) the new shaper prototype available at the CPFR.

Specifically, the “GRID” and “Planar” configurations refer to layouts featuring x‐y square holes and parallel stripes, respectively. (Figure 1).

Different field sizes ranging from 1 × 1 cm^2^ up to 5 × 5 cm^2^ are also available. In particular, the 5 × 5 cm^2^ field size was recently realized to enable the irradiation of larger area in vitro samples. Tungsten collimators with a central square hole for equivalent open field production are also available along with a new beam shaper device with variable sizes for in vivo experiments (Figure 1).

#### 2.1.1 Experimental setup @ CPFR

The new realized Planar1_3_5 and Planar1_2_5 minibeam collimators reported in Table 1 were characterized with the UHDR electron beams available at the CPFR, using EBT‐XD radiochromic films (RCF) placed perpendicularly to the beam, just after the minibeam collimator (no air gap). The procedure for film processing, analysis and calibration reported in^25^ was used to extract the transversal beam profile (scanning performed with DPI = 127 and the average dose with and without the minibeam template (equivalent open field). The results are reported in Section 3.

#### 2.1.2 Experimental setup @ Turin Linac

The *GRID1_2_2* collimator was also tested with the 10 and 18 MeV electrons accelerated at the Turin Linac. Specifically, the template was mounted just at the exit of the Linac head and the activation of the used metallic collimator was evaluated for 10 and 18 MeV electron beams. An increased non‐negligible activation at the higher available energies in Turin is expected and must be carefully evaluated from both radioprotection and patient‐safety perspectives. At 18 MeV, the harder bremsstrahlung spectrum includes a larger fraction of photons above the photoneutron production threshold, and the subsequent activation of heavy metals is expected to be more significant. A comparison of the gamma spectra produced after delivering a total dose of 20 Gy between 10 and 18 MeV electron beams and measured with a High Purity Germanium Detector (ORTEC) was performed. The data acquisition was conducted for 15 h right after the irradiation. Results are reported in Section 3.

#### 2.1.3 Experimental setup @ TPBL

Beside electron minibeams, a beamline for proton minibeam production was developed at the Trento Proton beam line (TPBL). Besides the realization of a novel dedicated facility for proton SFRT, the possibility to perform direct comparison with similar electron minibeam setups, thus emphasizing specific radiation quality dependence of the minibeam effect, was exploited. For this reason, an initial test campaign was performed at the Trento Proton beam line (TPBL) using the same *planar1_2_2* minibeam collimator listed in Table 1 and originally developed and used at CPFR with low energy electron beams.

**FIGURE 2:**
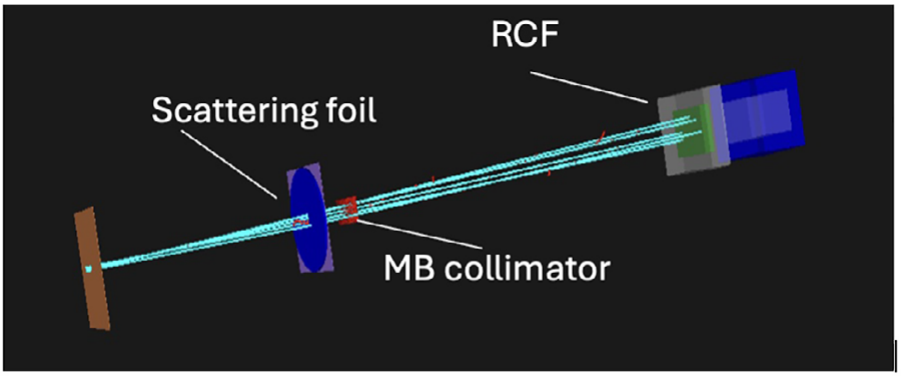
Scheme of the experimental setup employed at TPBL as simulated with TOPAS.

Specifically, the experimental setup employed is reported in Figure 2 showing the picture obtained from the Monte Carlo TOPAS simulations reproducing both the TPBL (uniform beam)^20^ and the minibeam setup. As shown in figure, the proton beam at the lowest energy available in the facility (70 MeV) traverses at first the scattering foil system currently employed in the facility to achieve a uniform beam for radiobiological experiments^20^ and, then, reaches the minibeam collimator *(planar1_2_2)*, placed at 1 cm of air from the scattering foil. EBT‐XD radiochromic films (RCF) were positioned perpendicular to the beam at the isocenter (7 cm from the minibeam collimator) to measure dose distributions. The experimental setup consisted of films stacked at different depths, separated by RW3 blocks.

A dedicated and systematic simulation work was then performed by simulating different collimator geometries in terms of: thickness, hole width, c‐t‐c distance and distance between the water phantom and the minibeam collimator. The results are reported in Section 3.

### 2.2 Dosimetric approaches and experimental setups

The accurate measurement of the wide range of dosimetric parameters involving the minibeam radiotherapy is one of the most critical and key steps towards the characterization and the reliable future use of MBRT in clinical practice. Moreover, the extreme spatial fractionation of the dose in MBRT requires the modification of the standard reference dosimetry protocols,^26^ also defining the reference conditions, that is, setup enabling the high accuracy absolute dose measurement. In MBRT, such reference conditions can be achieved by considering an open field (uniform dose distribution) equivalent to the minibeam field (same size). Under these conditions, standard dosimeters such as commercial ionization chambers can be used. However, the equivalent open field does not reproduce the characteristic minibeam pattern and therefore does not provide information relevant to minibeam irradiation.

On the other hand, the spatial features and the steep dose gradients of minibeam patterns, occurring at the millimeter scale, require the use of different dosimeters with the following characteristics:

- High spatial resolution or small sensitive volume (transversal size < 100 µm) to accurately reconstruct the minibeam parameters (peak dose, valley dose, PVDR distributions).
- Response unaffected by the lack of lateral charged particle equilibrium present under minibeam (non‐reference) conditions.
- Sufficient sensitivity to low doses (<1 Gy) in the valley regions.
- Energy‐independent response, since the energy spectrum varies across the minibeam pattern (between peaks and valleys) for both electron and proton minibeams.
- For charged particle beams with higher Linear Energy Transfer LET (e.g., protons), the capability to measure the LET variations across the minibeam pattern, through spatially resolved microdosimetric measurements. The beam quality expressed by the LET physical parameter is in fact directly correlated to the biological effect, that is, the RBE.
- Large‐area detectors (smaller than the total field size but large enough to include multiple minibeams) to evaluate average dose and average depth–dose distributions. This parameter is particularly relevant for fairly comparing minibeam with conventional uniform irradiations and correctly quantifying the differential biological effect.
- Dose‐rate independence, as the dose rate differs between peak and valley regions by a factor determined by the PVDR. This requirement becomes critical when combining minibeam irradiation with UHDR conditions.

In this context, the reference dosimeter for conventional RT, the ionization chamber, can only be used for average dose measurements. Radiochromic films certainly have already found applications, given their extreme spatial resolution. However, they are limited by their inaccuracy, operator dependence, energy spectrum dependences and time‐consuming data analysis.

The MIRO project approach relies on the developments of innovative complimentary enabling the real‐time measurement of the main dosimetric parameters supposed to be directly connected to the radiobiological outcome. Additionally, the MIRO project is also exploring the combination of UHDR and minibeams to study any possible synergy among the related biological effects. Therefore, the developed dosimeters will also be suitable for UHDR beams implying the possibility to also monitor the dose rate and its variation along the minibeam pattern. The different developed technologies are listed in Table 2 along with the corresponding dosimetric parameters and the details.

**TABLE 2:** List of the detectors developed within the MIRO project for reference, relative and in vivo dosimetry along with the features and references.

Detectors	Mode	Read out	Dose‐rate range	Time resolution	Beam parameter	Status
Micro SiC (100 um) multi‐detectors 100 um pitch	Real time, UHDR	Custom analog readout	up to MGy/s	<ns before integrator, 10 ms with integrator readout	PVDR, average dose	Electrons (in progress) and protons (future)
Large area (2.6 × 2.6 cm^2^) silicon sensor segmented in strips (180 um pitch)	Real time	Charge to frequency conversion ASIC (Tera09)	MGy/s (limited to kGy/s for simultaneous readout of 128 strips with present font‐end electronic	ns before front‐end readout, hundreds of us to s after front‐end readout	PVDR, average dose	Electrons^29^ and protons (in progress)
Thin 0.5 mm plastic EJ212 scintillator sheets	Real time imaging	Scientific camera	up to MGy/s	ns scintillation decay time, limited by camera frame rate (< 89.1 fps)	PVDR, average dose In vivo dosimetry	Electrons^27^ and protons (in progress)
Cerenkov luminescence emission in tissue	Real‐time surface imaging of the animal	Scientific camera	/	Prompt signal, limited by camera frame rate (< 89.1 fps)	In vivo dosimetry	In progress
Large 1 × 1 cm^2^ SiC detector	Real time, UHDR	Electrometer	up to MGy/s	< ns before integrator, 10 ms with integrator readout	Average dose PDD	Already characterized with electron minibeams

As shown in Table 2, active dosimeters are considered including both solid state detectors, that is, silicon strips and silicon carbide small and large area detectors, and organic scintillators both as sheets and fibers. Scintillators were already successfully characterized, and details of the technology as well as of the results can be found in.^27^

**FIGURE 3:**
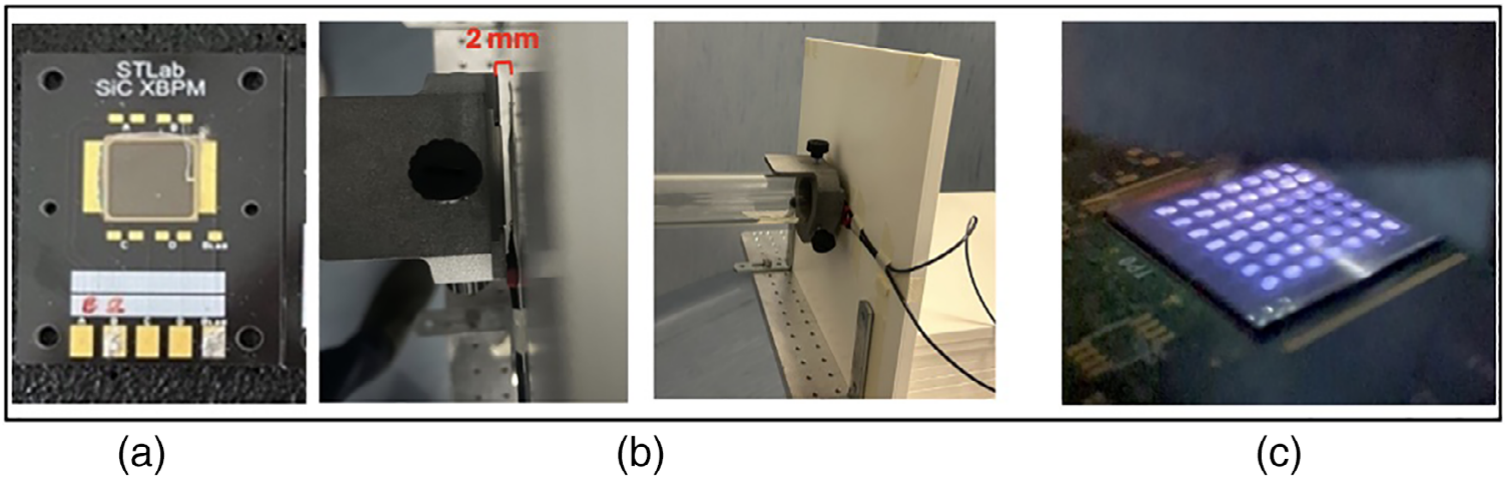
Pictures of the experimental setup employed at the CPFR for the irradiation of the large are SiC detector (a) with the minibeam template (b) placed at the end of the 40 mm diameter applicator. The SiC detector was placed as close as possible to the template (2 mm air gap) and aligned with the center of the beam. (c) Experimental setup employed at the Linac in Turin with the 2.6 × 2.6 cm^2^ strip silicon detector placed in air at 4 mm from the tungsten minibeam collimator.

The 1 × 1 cm^2^ 10 um thick PiN junction Silicon Carbide (SiC) detector listed in Table 1 was used for the real time measurement of the average dose of a minibeam pattern with the UHDR electron beams accelerated at the CPFR. As already demonstrated by Milluzzo et al.,^28^ such SiC detector is also suitable for the accurate dosimetry with UHDR beams showing a linear response up to an instantaneous dose rate of 3 MGy/s. The *Planar1_3_2, GRID1_3_2, Planar0.5_2_1, GRID0.5_2_1* minibeam collimators with the characteristics reported in Table 1, were attached at the end of the 40 mm diameter applicator as shown in Figure 3, while the detector was placed at about 2 mm of air from the surface of the minibeam collimator and aligned with the center. The produced charge in a single pulse (lasting 4 us) within the SiC detector active layer was measured using a Keithley 6517A electrometer, used also to supply a bias voltage of 200 V. The measured charge with the SiC detector for each set DPP can be converted to absorbed dose using the calibration factor (2.75 ± 0.06 Gy/uC) reported in^28^ obtained for the same SiC detector with the UHDR 9 MeV electron beam accelerated at the CPFR.

Open field square collimators were also used to measure the corresponding dose per pulse without minibeam pattern and calculate the ratio of DPP_mini/DPP_open.

A large‐area PIN silicon detector segmented into 128 strips each with an active area of 180 × 26.214 µm^2^ was also developed for the measurement of the transversal dose distribution at high spatial resolution.^29^ A preliminary test was carried out at the Turin Linac (Figure 3c) using the tungsten collimators developed at CPFR, namely, the configuration GRID1_2_2 described in Table 1. The silicon detector was placed in air at 4 mm from the minibeam collimator and a 200 V bias voltage was applied to deplete the layer up to 60 um. A custom multichannel readout ASIC, in conjunction with a National Instruments FPGA, was employed to simultaneously measure the charge deposited on each strip, thereby enabling the reconstruction of the projected profile of the minibeam grid. The reconstruction of the minibeam pattern subsequent to irradiation is currently the subject of ongoing investigations and a first preliminary result is reported in Section 3.

### 2.3 Radiobiological investigation of the minibeam effect

#### 2.3.1 Quantitative radiobiological advanced experiments

To characterize in‐vitro the radiobiological effects induced by minibeams, the MIRO project employs a multiscale experimental framework. This approach relies on both integrated response and local analyses of cellular responses to MBRT. To correlate the local cellular response with the spatial irradiation pattern, dedicated methodological pipelines are needed to spatially resolve localized effects, with the aim of highlighting also pattern‐dependent phenomena. Finally, complementary computational modelling strategies can be established to support and facilitate the interpretation of experimental data towards elucidating the underlying radiobiological mechanisms (see Section 3.3.2). To assess the effects of MBRT, we decided to use as first a lung in‐vitro model. Thus, the “overall” in‐vitro radiobiological effects induced by specific minibeam irradiation conditions on both cancer and healthy lung cells were evaluated in terms of survival fraction during a dedicated experiment carried out with the 9 MeV pulsed electron minibeams. Both conventional and UHDRs were available at the CPFR, to allow also the investigation of the combination of FLASH and minibeam effect in a controlled environment. In particular, human lung adenocarcinoma cells (A549) and human bronchial epithelial cells (16HBE) were irradiated using the following irradiation modalities:

- UHDR (FLASH): Average dose rate = 400 Gy/s, DPP = 1.3 Gy, Uniform beam
- CONV: Average dose rate = 6 Gy/min, DPP = 0.001 Gy, Uniform beam
- UHDR (FLASH) minibeam: integrated average dose rate = 400 Gy/s, integrated DPP = 1.3 Gy, planar1_3_2 minibeam collimator
- CONV minibeam; integrated average dose rate = 6 Gy/min, DPP = 0.001 Gy, planar1_3_2 minibeam collimator

A constant integrated average dose of 4 Gy for all the irradiation modalities was delivered in order to obtain a fair comparison between UHDR, conventional, UHDR minibeam, and conventional minibeam modes accordingly to previous data reported in.^30^

Beside taking the integrated average dose as a normalization point between the different irradiations, for the two UHDR and conv conditions (uniform and minibeam fields), also the integrated average dose rate (400 Gy/s and 6 Gy/min) and the integrated dose‐per‐pulse (1.3 Gy and 0.001 Gy) were kept constant to remove any possible dependence of the cellular response on these quantities (thanks to the capability and the versatility of the Linac at CPFR able to vary the beam current and all those parameters independently^18^).

**FIGURE 4:**
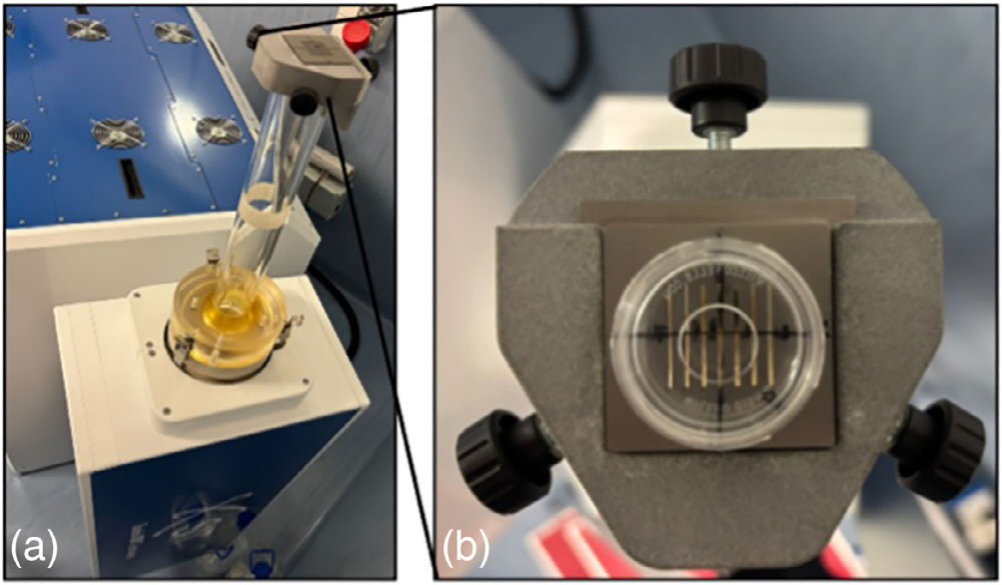
Pictures of the experimental setup employed at CPFR for the radiobiological experiments. The minibeam collimator was attached at the end of the 40 mm applicator displaced vertically (a), while the cells were placed within the plastic holder lied on top of the collimator (b).

The experimental setup is shown in Figure 4, the cells were placed at the bottom of multi well plate leaned on top of the minibeam collimator (*Planar*1_3_2), which was attached to the 40 mm diameter applicator displaced in vertical as shown in figure. The cells were then irradiated at a water depth of 1 mm, where a PVDR of about 20 was measured according to previously performed measurements.^21^

The survival rate 14 days after radiation exposure was evaluated observing the spatially integrated response to irradiation.

Clonogenic assay: to test cells’ clonogenic ability, after the irradiation, A549 and HB16 cells were plated in a 6‐well plate, at a concentration of 100 and 400 cells/well, for unirradiated control and 4 Gy administration, respectively, and cultured in a humidified environment at 37°C, 5% CO2. Clonogenic capacity was evaluated after 7 and 14 days, to enable the formation of the colonies, as previously described. Briefly, cell media was replaced every 3 days, and on the last day of incubation, cells were fixed in PFA 4% v/v (Sigma–Aldrich, St. Louis, Missouri, USA) in PBS and stained with crystal violet. Colonies with more than 50 cells were manually counted by the operators using the software ImageJ. The Surviving Fractions (SF) were calculated as reported by Franken and colleagues, and data were fitted using the linear‐quadratic model.

#### 2.3.2 Modelling

The use of the classical Normal Tissue Complication Probability (NTCP) models derived from the linear‐quadratic formalism to high PVDR minibeam radiotherapy is certainly limited. The direct radiation‐induced cell killing in valley regions in MBRT is likely influenced by non‐target effects and modulation of the vascular, immune and stromal microenvironment which are not considered in the current NTCP models.

**FIGURE 5:**
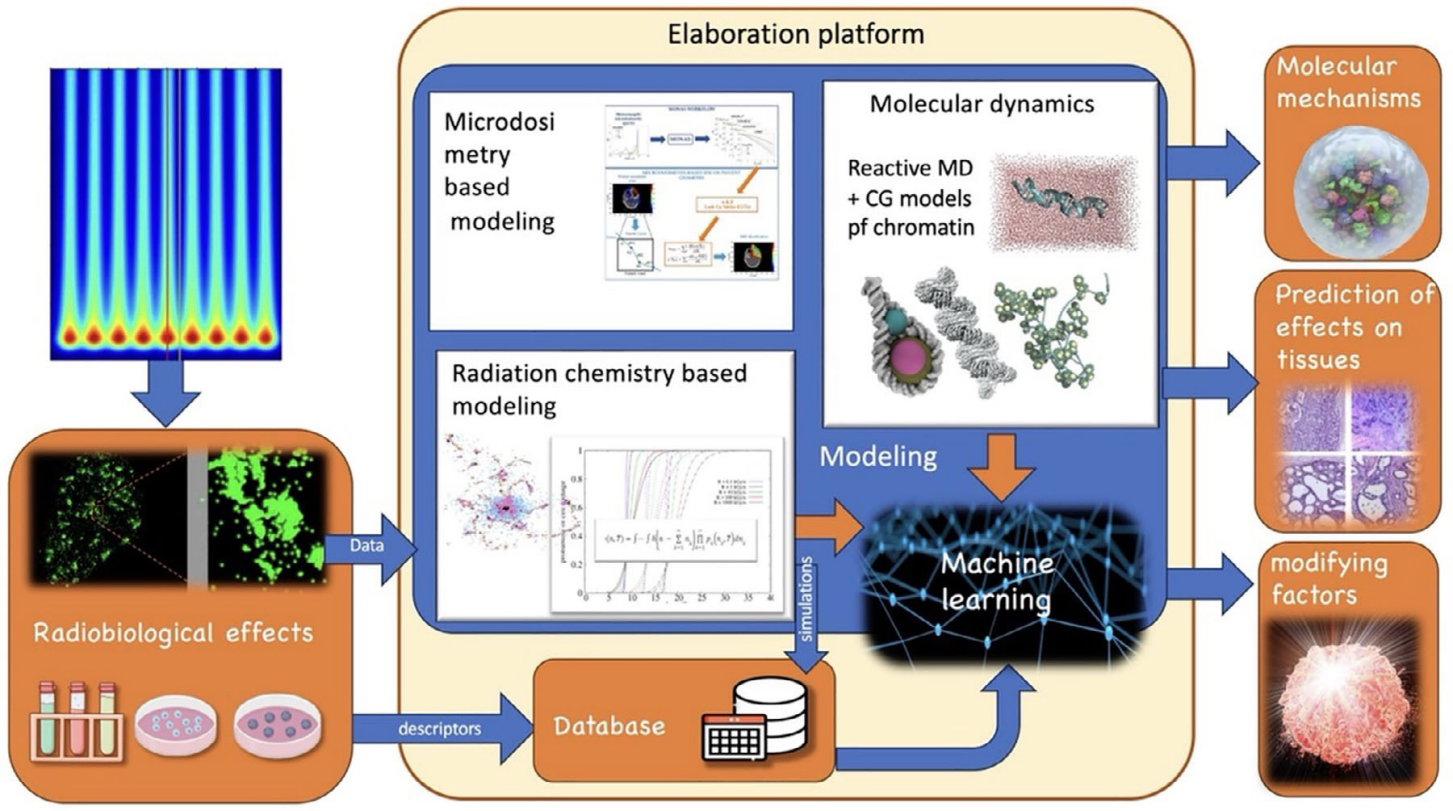
Schematic summary of the multiscale modelling workflow.

For these reasons, in parallel to experimental approaches, the minibeam effect has been investigated also with theoretical and simulation‐based methods with the aim to develop a more mechanistic NTCP framework that can incorporate the above mentioned, and usually missing, processes. In particular, several previous studies have been conducted, based on Monte Carlo (MC) simulations.^31^, ^32^ Most approaches were directed to explain the reduced toxicity in the normal tissue, while on the other hand, some experimental studies have resulted in observing also a higher killing effect on superficial tumors treated with a spatially fractionated radiation dose.^33^ Several works broadly explore different collimator configurations, which clearly impact not only the dose deposition pattern but also the related biological effects.^34^, ^35^ The MIRO approach, in this context, combines mechanistic multiscale modelling framework with AI based tool development.^36^ The initial phase of the project was dedicated to establishing a workflow scheme for the combination of modelling and simulation techniques with the aim of interpreting experimental data and elucidate the mechanisms of minibeam effectiveness. The workflow is schematically illustrated in Figure 5.

The experimental radiobiological data of different kinds, including super resolution images, are taken and either included in a database for further elaboration via ML techniques (see last paragraph) or directly used to compare with simulations and modelling. The modelling‐simulation tasks are separated basically in 4 types (included in the blue area in Figure 5), corresponding to the various steps of the modelling activities where research has been started:

1. Radiation chemistry
  Thanks to the development and experimental validation of the TRAXCHEM software^37^, ^38^, ^39^ for simulating the physico‐chemical stages of radiation‐induced chemical species, the model was optimized to represent the electron and proton beams used in the MIRO project's minibeam configurations for ultra‐high dose rate investigations. Evolution and recombination of radical species are modelled providing a spatiotemporally resolved framework for assessing the radiation chemistry effects across different regions of the irradiated field.
2. Molecular dynamics
  The molecular dynamics‐based modelling is used at different levels of representation to investigate the direct and indirect damage to DNA and its consequences.^40^ This approach enables simulations of systems large enough to include fully hydrated DNA fragments represented with atomistic detail. The reactions of DNA with different types of radicals and the impact of their spatial distribution can be investigated. Moreover, the mechanistic evolution of molecular damage in the DNA and the occurrence of double‐strand breaks over short timescales can be accurately simulated.
3. Microdosimetry based modelling
  Microdosimetry represents a very suitable framework to account for the inherent stochasticity in radiation quality^41^ minibeam irradiation field. Moreover, a multicellular extension of the Generalized Stochastic Microdosimetric Model (GSM^2^) for evaluating the impact of a spatially inhomogeneous irradiation pattern on a cell population has been developed, through an integration of GSM^2^ with an agent‐based cell population model. This model allows for the investigation of the biological effects of minibeams on highly proliferative cells, such as stem‐like cells, which are hypothesized to retain the capacity to repopulate peak‐dose areas, potentially influencing treatment outcomes. Importantly, the simulated microdosimetric quantities and the associated y_F_ and y_D_ values used for the GSM model to predict the biological effects among the peak and the valley, can be experimentally validated, using small size semiconductor (diamond/silicon/silicon carbide) microdosimeters having sensitive volumes smaller than both peak and valley widths. This enables direct measurement of microdosimetric spectra with sufficient spatial resolution to benchmark model predictions. ROS yields constitute an additional measurable endpoint through established chemical and fluorescence‐based assays. In^42^ we reported in silico comparisons of y_D_ and y_F_, and an experimental microdosimetry campaign for different minibeam configurations has already been completed, with results currently being prepared for publication. These datasets will provide the quantitative validation needed to further assess and refine the predictive performance of the framework.
4. AI‐based modeling
  Given the heterogeneity and variability of the experimental data related to the study of the minibeam effect, the development of a dedicated IT platform, an infrastructure based on the XNAT architecture, has been customized to support the joint management of imaging (e.g., fluorescence microscopy images) and tabular data (such as the different parameters characterizing the irradiation of the sample or the biological parameters) originating from diverse experimental contexts,^43^, ^44^The platform also supports the direct execution of AI‐based analysis on the data collected within the platform, for example, image analysis tools to accelerate and standardize the processing of fluorescence microscopy data.^45^ Moreover, machine learning algorithms, possibly combined with minimalistic empirical models^46^ can be trained on the collected data to predict the radiobiological effects of minibeam irradiation, providing interpretability and insights into the most relevant parameters driving the biological response.^47^
  We emphasize that this represents only the initial stage of a broader modelling effort: we are currently developing an extended framework incorporating cell migration, diffusion, and repopulation, to account for the potential contribution of valley region cells to repopulation and long‐term tissue sparing in MBRT.

### 2.4 Clinical perspectives

WP4 is developing a comprehensive simulation framework to assess the clinical feasibility and investigate the potential advantages of MBRT across different irradiation modalities, both alone and in combination with ultra‐high dose‐rate (UHDR) conditions. Within this framework, we explicitly distinguish between clinically realistic near‐term translation scenarios and longer‐term research developments enabled by emerging accelerator technologies.

Our approach focuses on clinical cases of interest (specifically those where conventional radiotherapy is limited) that could benefit from “minibeam” and “FLASH” effects. We generate virtual treatment plans optimized to reproduce spatial minibeam dose patterns, comparing them in terms of RBE with standard radiotherapy to evaluate their clinical potential. Near‐term clinical translation scenarios primarily include low‐energy electron and proton mini‐beam irradiations.

For superficial lesions, low‐energy electrons are considered due to their limited penetration depth. In this scenario, however, the minibeam pattern is delivered to both the lesion and adjacent healthy tissue; consequently, the biological advantage depends on achieving both healthy tissue sparing and isoeffectiveness on the tumor.

**FIGURE 6:**
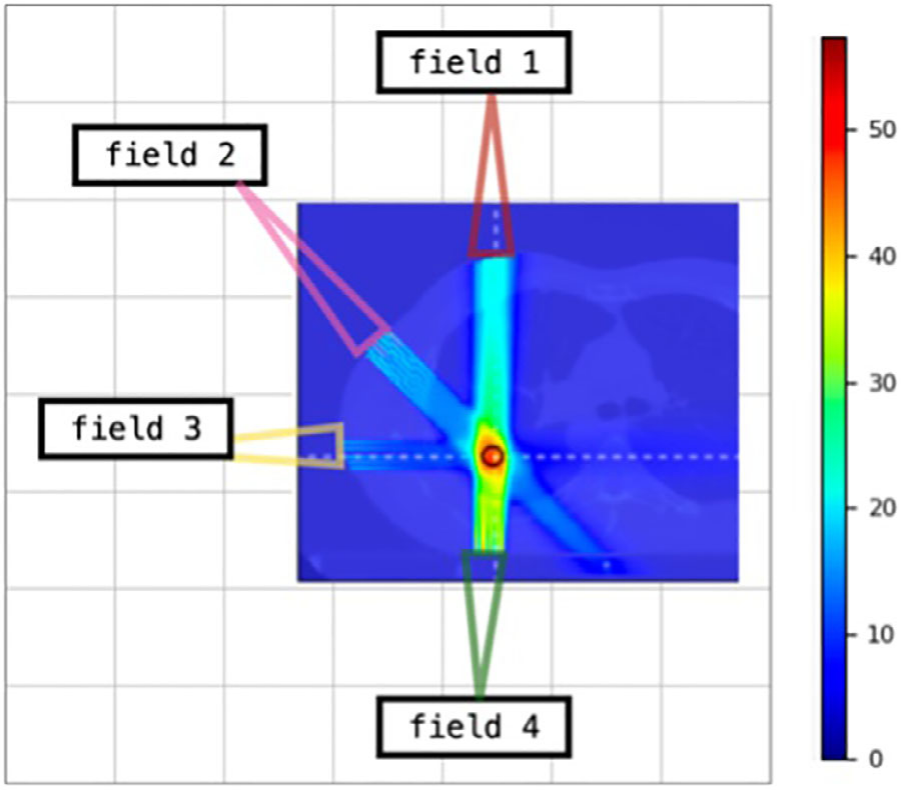
Illustrative example of the irradiation geometry adopted for preliminary minibeam VHEE simulations in non‐small‐cell lung cancer. The VHEE dose map is shown superimposed on the CT scan, with the PTV contours highlighted in black and the number of irradiation fields indicated. The dose distribution is shown for illustrative purposes only to visualize the beam geometry and mini‐ beam configuration, and does not represent a clinically optimized treatment plan.

For deep‐seated tumors, proton minibeam radiotherapy is investigated as a realistic clinical option. By combining the Bragg peak depth–dose distribution with spatial dose fractionation, proton minibeams allow the achievement of high peak‐to‐valley dose ratios (PVDRs) in superficial normal tissues and in organs located upstream of the tumor, while maintaining a homogeneous dose deposition within the planning target volume (PTV). This approach enables the preservation of the biological effectiveness of conventional proton therapy at the tumor level, as demonstrated in recent clinical.^48^ Long‐term research developments within WP4 focus on Very High Energy Electron (VHEE) minibeam irradiation for deep‐seated targets. VHEE irradiation emerges as one of the most promising candidates for combining minibeam spatial fractionation with UHDR dose‐rate conditions, as VHEE linacs are designed to intrinsically support the delivery of ultra‐high dose rates. This feature enables the simultaneous implementation of MBRT and FLASH radiotherapy within a single treatment scheme. Recent accelerator developments have already demonstrated the feasibility of VHEE minibeam scanning at FLASH‐relevant dose rates.^49^ In this context all computations are performed within a unified, high‐resolution Monte Carlo framework in which minibeam fields are generated, propagated and optimized in realistic anatomical geometries (see Figure 6). In the case of VHEE irradiation, simulations are performed assuming an IMRT‐like multi‐field geometry and a compact C‐band accelerator model capable of producing narrow, low‐divergence pencil beams with negligible energy spread. The beam model assumes millimetric to sub‐centimetric full width at half maximum (FWHM) at the exit window.^50^ Treatment plans are simulated using the GPU‐based fast Monte Carlo code FRED.^51^, ^52^, ^53^ After computing per‐electron dose maps, pencil‐beam fluences are iteratively optimized to satisfy plan dosimetric constraints. To assess FLASH‐related effects, voxel‐wise dose‐rate metrics are evaluated based on beam delivery parameters, and the biological dose is calculated by applying a FLASH modifying factor to the physical dose according to available experimental data.^54^ Building on the VHEE treatment‐planning algorithms previously developed and validated in earlier publications,^55^, ^56^, ^57^ we are currently developing a joint optimization framework that simultaneously accounts for both PVDRs and dose conformity. Through this integrated dosimetric–radiobiological approach, we aim to quantify peak–valley characteristics, target coverage, normal‐tissue sparing and FLASH‐mediated biological effects by systematically comparing minibeam and conventional irradiation strategies, with and without UHDR conditions.

## 3 RESULTS

### 3.1 Dosimetric characterization

#### 3.1.1 CPFR—electron minibeams up to 9 MeV

**FIGURE 7:**
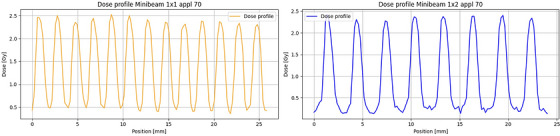
Transversal dose profile measured with the EBT‐XD RCF placed perpendicular to the beam direction at the entrance (depth = 0 mm) with 9 MeV electron beams at the CPFR using the planar1_2_5 (left) and planar1_3_5 (right) minibeam collimators.

The transversal dose profiles measured at the entrance with the RCF are reported in Figure 7 for the two minibeam configurations (*Planar1_2_5 and Planar1_3_5*).

The corresponding measured PVDR values are reported in Table 3 along with the corresponding ratio between the integrated dose measured over the ROI (region of interest) with (mini) and without (open) minibeam collimators. As expected, and in agreement with the previous results.^21^ Pensavalle et al., a much higher PVDR and a smaller ratio of the average dose resulted for larger c‐t‐c.

**TABLE 3:** Measurement of the ratio of the integrated dose obtained with the minibeam collimators (Dmini) and with the equivalent open field (Dopen) and the PVDR obtained for different collimator configurations with RCF and the SiC detector at CPFR.

	Configuration	Dmini/Dopen	PVDR
RCF	Planar1_2_5	0.52 ± **0.02**	5.50 ± **0.27**
	Planar1_3_5	0.34 ± **0.02**	14.71 ± **0.73**
1 × 1 cm^2^ SiC	Planar1_3_2	0.31 ± **0.01**	/
	GRID1_3_2	0.22 ± **0.01**	/
	Planar0.5_2_1	0.16 ± **0.01**	/
	GRID0.5_2_1	0.08 ± **0.01**	/

**FIGURE 8:**
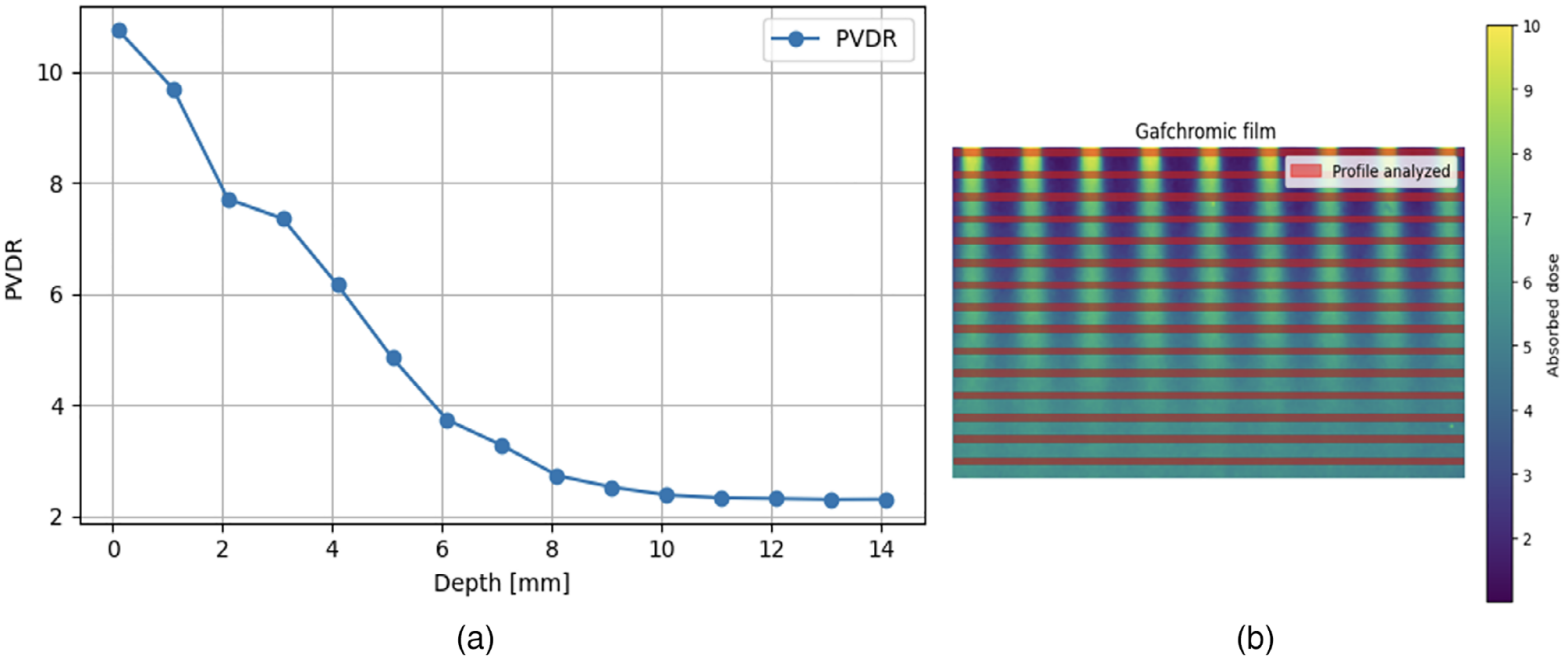
(a) PVDR as a function of the depth in water measured with the EBT‐XD RCF placed parallel to the beam direction at the CPFR using the planar1_3_5 (left) minibeam collimator. (b) The dose map resulting from the RCF analysis along with the depths where the PVDR was calculated is also reported.

The PVDR values were also measured at different water depths for the *planar1_3_5* collimator (Figure 8a) placing the EBT‐XD RCF parallel to the beam direction (among two PMMA slabs) and sampling the PVDR at different positions as shown in Figure 8b. A “minibeam” region (PVDR > 2.5) up to a water depth of 9 mm is obtained.

Such measurements are essential to correlate the PVDR estimated at the entrance (z = 0) and the one at the real position of the cells or sample to irradiate. For instance, for the in vitro measurements here reported, the cells were positioned on top of a 1 mm water‐equivalent flask slide, corresponding to an effective water depth of z = 1 mm rather than z = 0. The measurements shown in Figure 8 which need to be performed for the specific minibeam collimator employed, enable to obtain the real PVDR at the cell position, which is reduced approximately of 30% as respect to the value at the entrance (for this configuration).

For more complex situations, such as future in vivo irradiations where different tissue types traversed by the radiation beam must be considered and may not be water‐equivalent, we have developed a Monte Carlo simulation code capable of realistically reproducing the electron FLASH linac, including the minibeam collimators attached to the applicators. The code also incorporates patient CT anatomical images in order to calculate the realistic dose distributions.

The ratio between the integrated dose per pulse measured with the SiC detector for the four minibeam configurations and the corresponding value obtained with the open field is also reported in Table 3. These values have been averaged over the different dose per pulses (beam current) set during the measure, that is, from conventional to the UHDR regime. The uncertainties reported in Table 3 are arising from the uncertainty on the dose calibration for the SiC detector (around 2% as reported in^17^) and for the RCF (around 5% as reported in^14^). As shown, the ratio varies from 0.31 down to 0.08, where both the hole width and the c‐t‐c distance were varied.

Planar1_3_2 and GRID1_3_2 collimators have been already characterized using radiochromic films in^21^ in terms of PVDR and Dmini/Dopen resulting of 0.31 and 0.22, respectively. The results shown in Table 3 and obtained with the large SiC detector are in agreement with both the previous measurement performed with the radiochromic films and with the ones obtained through the Geant4 Monte Carlo simulations^22^ for the same minibeam collimator patterns.

 were already characterized with radiochromic films, are perfectly in agreement with the previous work reported in^21^ and with the ones obtained through Geant4 Monte Carlo simulations^22^ for the same minibeam collimator patterns. The *eFLASH_radiotherapy* Geant4 advanced example realistically simulating the ElectronFLASH linac installed at the CPFR was used for simulations. The application, publicly distributed in the official Geant4 release, reproduces the realistic dose distributions with and without the minibeam collimators as previously validated with experimental data.^58^

Similar results were also found using scintillators at the same experimental conditions, as already reported in.^27^ As shown, the measured FWHM and c‐t‐c distance matched the reference values that were deduced from the specifications of the tungsten collimators. Therefore, it was demonstrated that the scintillator sheet allowed for a clear reconstruction of the minibeam dose profiles.

#### 3.1.2 Turin Linac—electron minibeams up to 18 MeV

**FIGURE 9:**
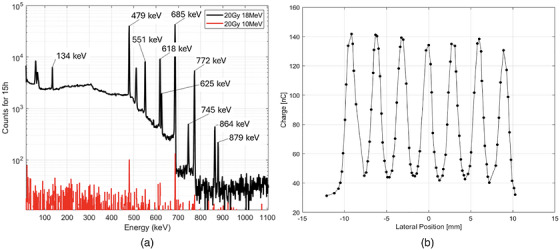
(a) Gamma‐ray spectra of tungsten collimators irradiated at 18 MeV (black) and 10 MeV (red) at the Linac in Turin, acquired over 15 h. (b) 10 MeV electron minibeam profile obtained with the silicon strip detector and the GRID1_2_2 collimator at the Turin facility.

Figure 9a shows the resulted gamma ray energy spectra measured after the irradiation with 10 MeV (red lines) and 18 MeV (black lines) electron beams.  The background contribution was subtracted using measurements acquired from a non‐irradiated collimator. The result highlights that a single delivery at 18MeV resulted in an evident activation of the collimator material with the main peaks corresponding to the 187 W identified after a comparison with the gamma spectroscopy database,^59^ while only the main peaks are visible for the 10 MeV sample. Given that the collimators are composed of ASTM B760 tungsten (93%), with minor amounts of nickel (4%) and copper (3%), the increased counts at 511 keV at the highest electron energy can be attributed to the β^+^ decay of 57Ni, expected to be produced at energies above approximately 12 MeV.

Figure 9(b) shows the transversal distribution measured with the large area Silicon strip detector using the *GRID1_2_2* minibeam. As shown, the integrated measured charge varies from values of about 45 nC up to 140 nC revealing a measured PVDR of about 3.2 at the surface. This result underestimate the value of the expected PVDR, which is about 8 as it is worth considering that the signal of each peak in Figure 8(b) integrates the measurements of the different holes peaks along the corresponding sensor strip. The measurement conducted here is a projection in one direction of the minibeam field, resulting in a smaller PVDR than the expected one. Therefore, tomographic reconstruction is being considered to accumulate the information from different angles.

#### 3.1.3 TPBL—proton minibeams up to 70 MeV

**FIGURE 10:**
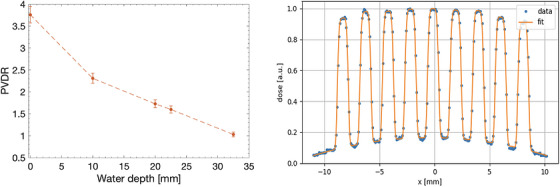
(a) PVDR measured with the RCF stack at five different water depth (b) Lateral (x) normalized dose profile in arbitrary units [a.u] measured at the entrance (0 mm) with the RCF along with the multiple gaussian fit performed over the data.

Figure 10: on the left, PVDR measured with the RCF stack at five different water depth; on the right, lateral (x) normalized dose profile in arbitrary units [a.u] measured at the entrance (0 mm) with the RCF along with the multiple gaussian fit performed over the data using 70 MeV proton minibeams.

A multiple generalized gaussian fit, defined as $Dose=\sum_{i=1}^{9}A_{i}e^{-{\left(\frac{{\left(x-\mu_{i}\right)}^{2}}{2\sigma_{i}^{2}}\right)}^{P}}$, was performed over the transversal dose distribution shown in Figure 10 (right), where A_i_, m_i_ and s_i_ are, respectively, the normalized amplitude, the position and the standard deviation of the i‐th minibeam peak. The parameter *P* accounts for the fact that the shape of the single peak is not perfectly Gaussian as the distribution appears flatter around the center due to the non–point‐like nature of the source.

The results of the fitting procedure, the parameters are reported in Table 4.

**TABLE 4:** Resulting fitting parameters for the dose distribution shown in Figure 10b.

	1	2	3	4	5	6	7	8	9
A [a.u.]	1.94	1.92	1.90	1.90	1.87	1.90	1.91	1.94	1.88
m [mm]	−7.92	−5.84	−3.76	−1.67	0.41	2.50	4.58	6.67	8.74
σ [mm]	0.59	0.60	0.60	0.59	0.60	0.59	0.61	0.59	0.59
P	−1.79 10^−3^								

However, considering the values obtained in the literature^23^ with similar setups, a further optimization especially of the collimator geometry, is certainly required; this is being conducted through Monte Carlo simulations.

**FIGURE 11:**
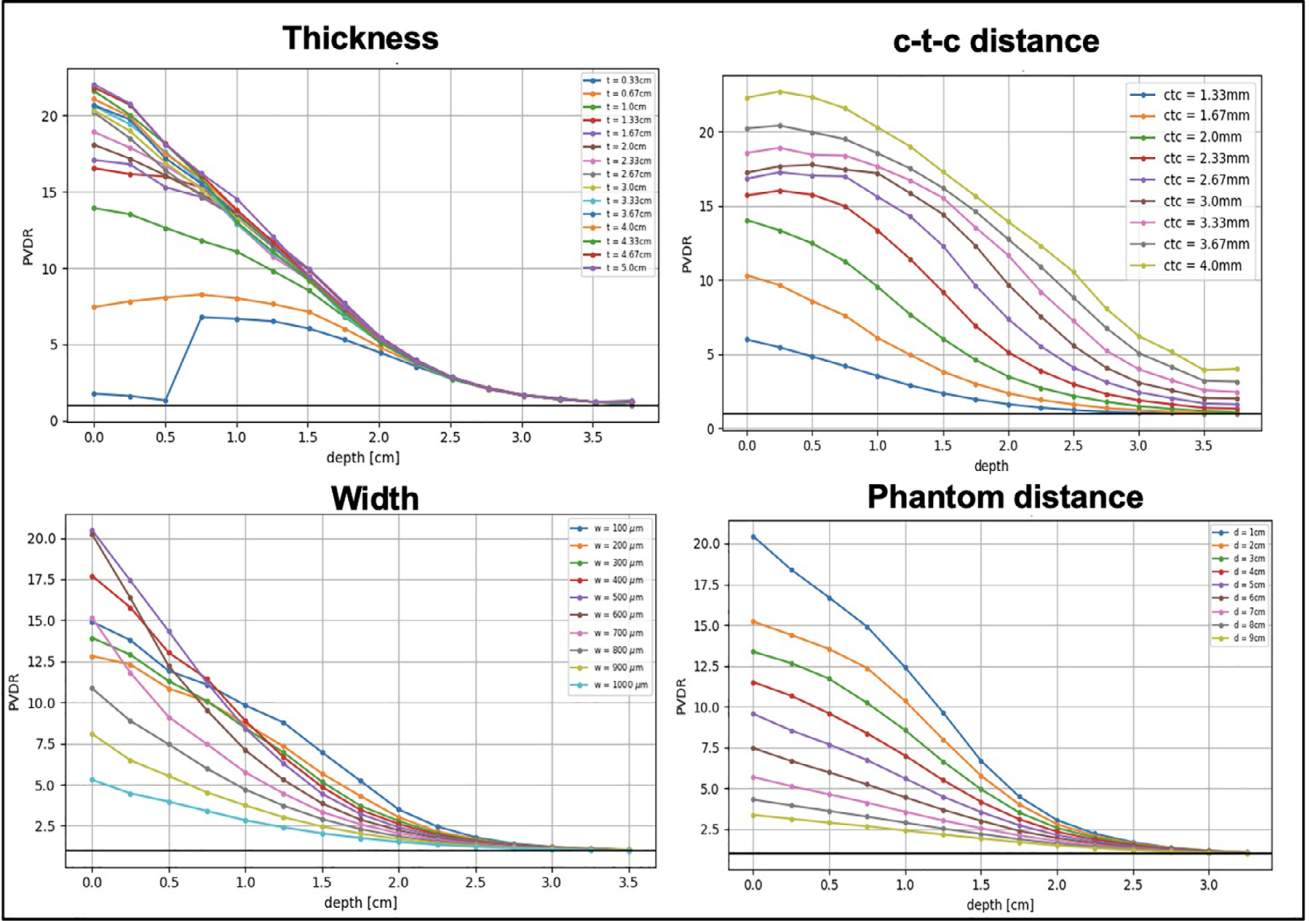
PVDR distributions in water depth, corresponding to varying parameters, respectively, in thickness, center to center distance, hole width, and distance of the phantom to the beam exit.

The PVDR values as a function of the water depth resulted from the simulations, varying the different parameters, are reported in Figure 11. The collimator tungsten thickness was varied from 0.33 cm up to 5 cm, the c‐t‐c distance from 1.33 mm up to 4 mm, the hole width from 100 um up to 1 mm and the phantom distance from 1 cm up to 9 cm. The results in terms of highest PVDR and minibeam penetration capability, returned a clear advantage in using the thicker collimators (5 cm), the highest c‐t‐c distance (4 mm), a hole width of 500 um and a phantom distance of 1 cm (the minimum). On the other hand, the beam current losses have been evaluated to be critical for some conditions, thus a compromise must be found.

The reduction in overall flux obtainable with the passive scattered setup employed, may indeed reach up to 3 orders of magnitudes and thus approaching a region of dose rates which would make experiments unfeasible. Given a future possibility on the TPBL to install a pencil beam scanner coupled to a passive minibeam collimator, this limit could be importantly overcome.

### 3.2 Radiobiology of the minibeam effect

#### 3.2.1 First in vitro radiobiological results with UHDR electron beams

**FIGURE 12:**
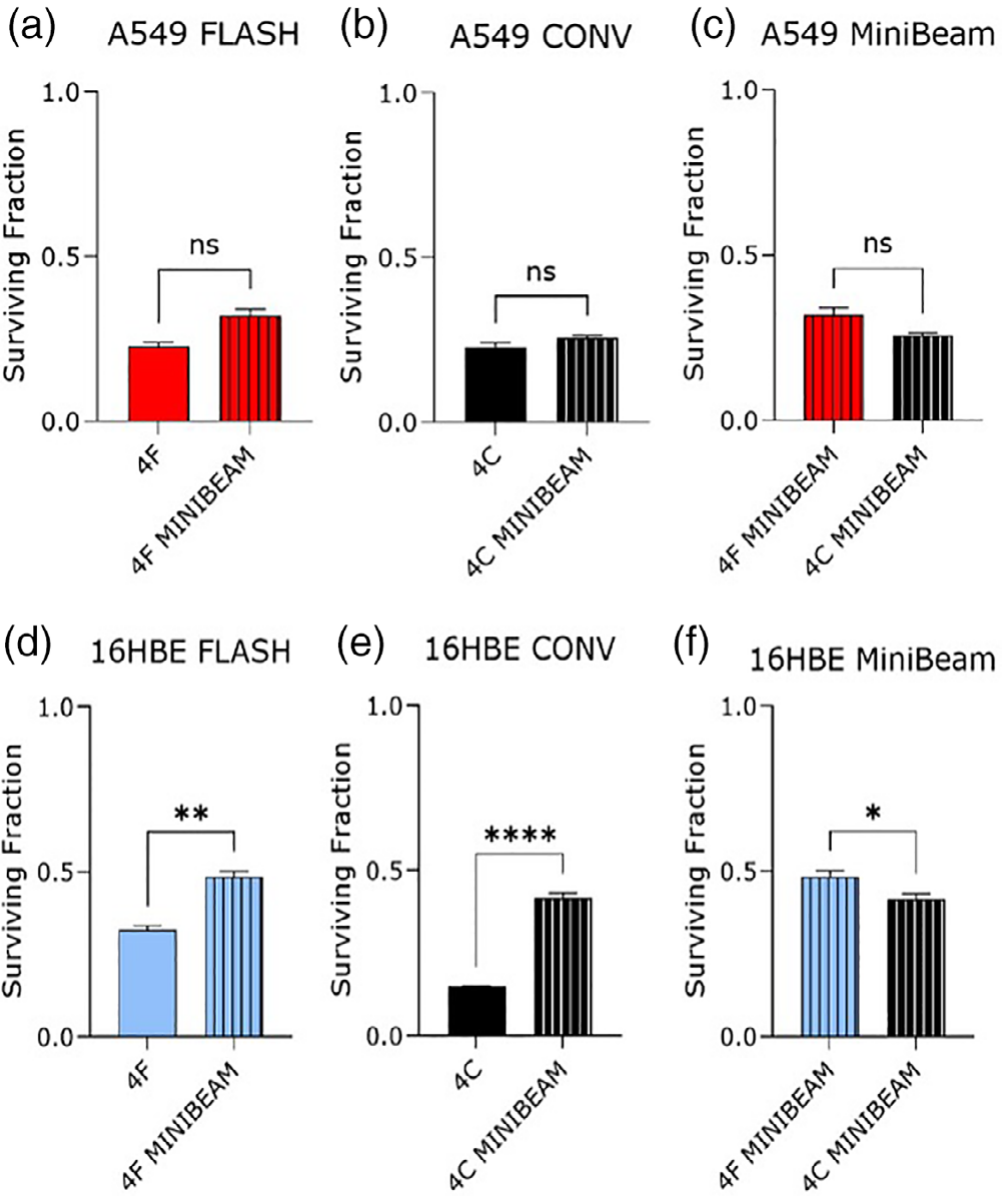
Panels from A to C show the surviving fraction of tumoral cells; panels from D to F show the surviving fraction of healthy cells. Panels A and D report the comparisons between open field and minibeam with UHDR beams (FLASH‐RT), while panels B and E show the comparisons in CONV modality. Panels C and F are related to comparison between FLASH and CONV effects using minibeam template during the irradiation. Data are reported as mean ± SEM. Results, from *N* = 3 independent replicates, are reported in Table 3 and statistical analyses were performed using One‐Way ANOVA followed by Dunnett's multiple tests. Diagrams are normalized on the plating efficiency of the untreated control and the number of cells seeded in each treatment group. *p*‐values: (a) ns: *p* = 0,057; (b) ns: *p* = 0,1999; (c) ns: *p* = 0,099; (d) **p = 0,002; (e) ****p < 0,0001; (f) *p = 0,047.

The clonogenic assay, that is, the ability of cells to form colonies, was performed for both the A459 cancer and 16HBE healthy cells. As shown in Figure 12, for A549 cells we observed a statistically significant reduction in surviving fraction with respect to control, both for open field and for Minibeam irradiation, in FLASH and CONV modalities. Regarding the 16HBE healthy cells, a similar reduction of survival was evidenced and unfortunately, the irradiation at CONV open field registered the worst survival index (*p* < 0.001). On the other side, comparing the open field technique with the minibeam one (Figure 12a,b,d,e), we found comparable results in terms of killing lung tumoral cells which could appear to be isoeffectiveness of the two modalities, and different effects on healthy cells. Indeed, data, divided into two different graphs based on irradiation FLASH or CONV modalities, evidenced for the tumoral cell line that all treatments were equally effective, and no significant differences were found between the types of treatment (Figure 12a,b). Otherwise, in healthy cells, the minibeam technique seems to permit an increase of surviving fraction, in respect to open field (Figure 12d,e); notably the sparing effect is most evident in CONV modality (*p* < 0.0001). Lastly, a statistical analysis of the comparison between the effects obtained after FLASH or CONV irradiations using minibeam template was performed; the results evidenced that in tumoral cells no significant differences were found between the two irradiation modalities (Figure 12c). In healthy cells, the comparison between minibeam FLASH and minibeam CONV showed a significant increase in cell survival when exposed to the FLASH regime with the template (*p < 0.05) (Figure 12f).

A more detailed understanding of the radiobiological effects induced by MBRT can be obtained by correlating the DNA damage^60^ with the spatial position of individual cells within the irradiation pattern and the corresponding local dose distribution. However, a standardized strategy for correlative RT and super‐resolution imaging is missing. To address this, a correlative imaging workflow based on fluorescence super‐resolution microscopy^61^, ^62^ was developed, specifically designed to assess DNA damage at the single‐cell level in relation to the local irradiation dose within the MBRT pattern. In this perspective, the correlative method for SR developed so far represents a robust tool for better elucidating the relationship between the irradiation parameters and radiobiology in SFRT, with subsequent progress in unveiling its full potential.

Results, from *N* = 3 independent replicates, are reported in Table 5 and statistical analyses were performed using One‐Way ANOVA followed by Dunnett's multiple tests (See Table 5).

**TABLE 5:** Statistical analyses were performed using One‐Way ANOVA followed by Dunnett's multiple tests and experiments are based on *N* = 3 independent biological replicates; Results are reported as mean ± SEM and standard deviation is calculated for the different irradiation conditions.

	16HBE			A549		
	Mean ± SEM	Std Dev	N. ind	Mean ± SEM	Std Dev	N. ind
CTRL	1.00 ± 0.03	0.06	3	1.00 ± 0.07	0.09	3
4F Open field	0.32 ± 0.01	0.02	3	0.227 ± 0.011	0.02	3
4F MINIBEAM	0.48 ± 0.02	0.03	3	0.228 ± 0.013	0.02	3
4C Open field	0.15 ± 0.001	0.002	3	0.32 ± 0.02	0.03	3
4C MINIBEM	0.42 ± 0.02	0.03	3	0.26 ± 0.01	0.01	3

## 4 DISCUSSION AND FUTURE WORK

As above mentioned, the MIRO project aims at systematically studying the dependencies of the minibeam effect from the physical quantities, that is, the dose distributions as well as the geometrical pattern parameters, the particle type (electrons or protons) and the energy distribution.

Three experimental platforms were setup within the project and are currently available for experiments with minibeams, including low energy electrons (CPFR), medium energy electrons (Turin) and high energy proton minibeams (Trento Protontherapy Centre). Specifically, the CPFR, where 9 MeV UHDR pulsed electron minibeams are produced, also enables the systematic study of the possible synergistic effect of the minibeam and the FLASH effect. Thanks to the triode gun, the beam current within the single pulse can be varied, giving the possibility to normalize not only for the same integral dose but also for the same integral average dose rate and the integral dose‐per‐pulse, both necessary conditions for a fair comparison of an UHDR minibeam field with UHDR uniform field (conventional) irradiations.

The higher energy electrons (18 MeV) available at the Turin linac will also serve to evaluate the dosimetric challenges at higher energy, as for instance the activation of the metallic collimators used for the minibeam production, which could become relevant in the perspective of VHEE beams. Finally, the proton minibeams available at the Trento Protontherapy Centre will allow the comparison with the experimental results presented in the literature, mainly obtained with proton minibeams. Por the project this will be a fundamental term of comparison for the results obtained with the never explored electron minibeams.

Accurate and innovative dosimetric systems are being developed to enable a comprehensive characterization of minibeam dose patterns. These systems are coupled with validated Monte Carlo simulation codes, which serve as reference tools for precise radiobiological experiments and support the future clinical implementation of Minibeam Radiotherapy.

The use of a validated Monte Carlo code reproducing the experimental setup will allow the retrieval of the 3D dose distribution and the prediction of the integrated average dose for both the minibeam configuration and the corresponding open‐field irradiation. As discussed previously, a fair comparison between minibeam and uniform irradiation requires the integrated average dose to be kept constant; therefore, it must be accurately calculated or experimentally measured.

The radiobiological investigation of the minibeam effect within the MIRO project combines experimental studies to theoretical approaches based on multiscale modelling. The experiments investigate both the effect at the microscopic scale and, using advanced high‐resolution microscopy techniques, the damage dose‐pattern correlation. The modelling, working in continuous feedback with experimental data, provides hypotheses on the underlying mechanisms and suggests possible new experiments.

As a first approach, a radiobiological in vitro study was performed using the 9 MeV electron minibeams available at the CPFR. The clonogenic assay, which is the gold standard in radiobiology research, was used as endpoint to quantify the fraction of cells capable of maintaining reproductive integrity after different irradiation conditions.

Although the study is limited to quantify the total integral damage (through survival fraction evaluations) induced by minibeam irradiation to both tumoral human lung adenocarcinoma cells (A549) and healthy human bronchial epithelial cells (16HBE), the results highlight the different survival fraction induced by the different irradiation modalities employed, that is, minibeam + CONV rate, uniform + CONV rate, uniform + UHDR and the combination of minibeam + UHDR. Specifically, the minibeam + UHDR modality results in an enhanced survival fraction for healthy 16HBE cells compared to both the minibeam + CONV and uniform + UHDR modalities. Despite the in vitro nature of the study, these findings suggest a possible synergistic effect between the two irradiation techniques. While in vitro results are inherently limited and not directly translatable to clinical practice, they offer valuable insights into the underlying radiobiological mechanisms. Furthermore, these findings can guide future in vivo studies, ensuring they are restricted to those strictly necessary, a crucial consideration for ethical, financial, and time‐related reasons.

Beyond the lung cancer model presented in this study, future research will extend to breast cancer (BC) cells, particularly triple‐negative subtypes, which remain among the most aggressive and treatment‐resistant malignancies. Building on preclinical evidence showing improved doxorubicin delivery facilitated by MiniBeam irradiation in triple‐negative BC (TNBC) mouse model^63^ our ongoing work focuses on elucidating minibeam‐induced oxidative stress and its impact on tumor biology. Reactive oxygen species (ROS) are well‐established mediators of radiation response; however, their modulation following spatially fractionated irradiation (i.e., minibeam RT) remains poorly characterized despite its relevance. Mechanistically, it is demonstrated that MBRT downregulates VEGFR (Vascular Endothelial Growth Factor Receptor) signaling, alleviating hypoxia and angiogenesis, and enhanced vascular normalization via increased pericyte coverage.^63^ Using the highly tumorigenic MDA‐MB‐231 TNBC cell line, preliminary data suggest that minibeam irradiation enhances intracellular oxidative stress, reflected by a decreased GSH/GSSG ratio, and this effect appears independent of dose or beam delivery schedule (data not shown). These findings highlight the need to investigate how oxidative stress influences DNA damage kinetics and repair processes, which may contribute to genomic instability and therapeutic response. Future investigations focusing on the kinetics of DNA damage induction and repair in BC cells will be crucial to establish a mechanistic link between the temporal evolution of oxidative stress and the dynamics of genomic instability following minibeam irradiation.

Moreover, a mechanistic interpretation of the sparing effect observed in minibeam irradiation can be framed within the classical Functional Subunit (FSU) paradigm, widely adopted in clinical and preclinical radiobiology.^64^ In this view, each organ is modeled as a collection of FSUs characterized by a seriality index, and organ dysfunction emerges when a sufficient fraction of FSUs is inactivated. The spatially modulated structure of minibeams fits naturally within this framework, as valley regions preserve a portion of FSUs that can compensate for those lethally damaged in the peaks. Beyond this geometric sparing, minibeam irradiation may also trigger additional biological mechanisms. The presence of viable tissue and microvasculature in valley regions can support repopulation, diffusion mediated repair, and potentially differential ROS signaling between peaks and valleys. These processes may facilitate recovery of peak damaged areas and contribute to the enhanced tissue tolerance observed in MBRT. The model proposed within the MIRO project captures part of this behavior through the seriality dependent volume effect, and ongoing work aims to integrate explicit repopulation and diffusive repair mechanisms into a more comprehensive mechanistic NTCP framework.

Regarding the future in vivo studies, considering the limited penetration of the low energy electron minibeams currently available in the project, we will focus the experiments on the skin irradiation, which, in addition to being an organ of extreme interest from a radiobiological point of view, also represents an organ at risk from any type of radiotherapy treatment.

Based on the distinct oxidative and metabolic responses observed in B16F10 murine melanoma cells following UHDR irradiation compared to CONV irradiation (preliminary data, not shown), we are investigating whether minibeam administration reproduces or enhances these tumor‐selective effects. Specifically, we will evaluate whether minibeam modulates ROS dynamics, mitochondrial respiration, and ATP production in B16F10 cells while preserving metabolic function in normal melanocytes.

Looking ahead, a subsequent in vivo pilot study in the B16F10 melanoma mouse model will further determine whether these molecular and metabolic signatures translate into improved tumor control, enhanced cytotoxicity, and sparing of surrounding healthy tissue.

Finally, we have developed a series of simulation tools capable of creating virtual minibeam and UHDP treatment plans, varying the beam type (protons and electrons), energy (low energy and VHEE), and emission mode (broad field and pencil beam scan) in the case of electrons. As shown in the results, we are already able to produce dose distributions for optimized plans and compare them with conventional treatment plans. The next step, which will allow us to evaluate the true clinical potential of these new methods, is to use Dose‐modifying factors (DMFs) that will be provided by experimental data (ours and those of other research groups). The comparison between plans will be made in terms of overall radiobiological effectiveness (RBE).

## CONFLICT OF INTEREST STATEMENT

M. Camarda is the STlab founder. J. Pensavalle and G. Felici are SIT S.p.A. employees.

## Data Availability

Authors will share data upon request to the corresponding author.
